# A randomized phase 1b/2 trial of ABBV‐916 in adults with early Alzheimer's disease

**DOI:** 10.1002/alz.71715

**Published:** 2026-08-07

**Authors:** Shau Yu Lynch, Yamin Wang, Deli Wang, Sagar S Bachhav, Hao Xiong, Joey Boiser, Edwin Stage, Anthony W Bannon, Ole Graff, Hana Florian

**Affiliations:** ^1^ Clinical Development AbbVie Inc. North Chicago Illinois USA; ^2^ Statistics AbbVie Inc. North Chicago Illinois USA; ^3^ Clinical Pharmacology AbbVie Inc. North Chicago Illinois USA; ^4^ Global Patient Safety AbbVie Inc. North Chicago Illinois USA; ^5^ Discovery Science AbbVie Inc. North Chicago Illinois USA; ^6^ Precision Medicine AbbVie Inc. North Chicago Illinois USA

**Keywords:** amyloid, amyloid‐related imaging abnormality (ARIA), apolipoprotein E ε4 allele (APOE ε4), amyloid beta 42/40 (Aβ42/Aβ40), AβpE3, clinical trial, glial fibrillary acidic protein (GFAP), multiple ascending dose, pharmacokinetics, phase 1b, positron emission tomography (PET), p‐tau217

## Abstract

**INTRODUCTION:**

ABBV‐916, an anti‐amyloid immunotherapy, was evaluated in patients with early Alzheimer's disease (AD).

**METHODS:**

This phase 1b/2, double‐blind, placebo‐controlled study consisted of two stages: multiple ascending dose (Stage A) and dose expansion (Stage B). In Stage A, patients were randomized to one of six planned cohorts to receive intravenous ABBV‐916 (10 mg to 3000 mg) or placebo monthly through week 24. Assessments included amyloid PET, blood‐based biomarkers, pharmacokinetics (PK), and safety. Dose selection for Stage B was to be based on results from Stage A.

**RESULTS:**

One hundred six patients were randomized to ABBV‐916 or placebo. The 3000 mg dose was reduced to 2000 mg and subsequently to 900 mg after safety review. Dose‐proportional PK were observed, and amyloid reduction was observed over 24 weeks at doses ≥300 mg. Most adverse events were non‐serious amyloid‐related imaging abnormalities. The program ended before dose expansion due to business considerations.

**DISCUSSION:**

Amyloid clearance rate and safety of ABBV‐916 were generally comparable to approved anti‐amyloid AD therapies.

**CLINICAL TRIAL REGISTRATION INFORMATION:**

NCT05291234

## Highlights

- This was a phase 1b/2, randomized, double‐blind, clinical trial of ABBV‐916.
- ABBV‐916 at 300 and 900 mg Q4W over 24 weeks of treatment demonstrated the potential to reduce brain amyloid plaques at rates comparable to currently approved anti‐amyloid therapies.
- ABBV‐916 exhibited dose‐proportional PK, with safety comparable to approved anti‐amyloid AD therapies.
- We report the amyloid clearance rate and safety outcomes over 24 weeks of treatment.

## 1 BACKGROUND

Of the more than 57 million people estimated globally to have dementia, Alzheimer's disease (AD) may be responsible for 60% to 70% of cases.^1^ The prevalence of AD is only expected to increase as the population ages.^2^ Although the cause of AD is unknown, a pathological hallmark of AD is the accumulation of amyloid beta (Aβ) plaques in the brain, formed by the aggregation of Aβ peptides.^3^, ^4^ These plaques are associated with early AD pathogenesis and may eventually lead to the cognitive decline typically observed in patients with AD.^2^, ^3^, ^5^ Aβ plaques and other pathological changes can be widespread throughout the cortex years before clinical symptoms emerge.^5^, ^6^

Recently, several disease‐modifying, amyloid‐targeting therapies have been approved for the treatment of AD.^7^, ^8^ Currently approved symptomatic and disease‐modifying therapies for AD have demonstrated modest clinical benefits.^9^, ^10^, ^11^ Additionally, Aβ‐directed monoclonal antibodies carry boxed warnings for amyloid‐related imaging abnormalities (ARIA), a class of adverse events (AEs) involving cerebral edema (ARIA‐E) or hemorrhage (ARIA‐H).^7^, ^8^, ^11^ The risks of ARIA are also influenced by apolipoprotein E (*APOE*) genotype, with *APOE* 𝜀4 carriers having a higher risk of developing ARIA than non‐carriers.^12^ Consequently, there is an unmet need for more efficacious and tolerable disease‐modifying agents.

ABBV‐916 is a recombinant humanized immunoglobulin G1 monoclonal antibody that selectively binds Aβ peptides bearing a pyroglutamate residue at amino acid position 3 (AβpE3) and is being investigated as a potential disease‐modifying treatment for AD. As AβpE3 plaques are less abundant in the cerebral vasculature, ABBV‐916 may have less potential for ARIA. ABBV‐916 showed evidence of amyloid clearance without microhemorrhage in preclinical studies.^13^ In a single ascending dose (SAD) study, ABBV‐916 doses up to 3000 mg were well tolerated in healthy participants.^14^ No clinically significant laboratory findings, ARIA, or serious adverse events were reported. The ABBV‐916 pharmacokinetic (PK) profile exhibited dose‐related increases in maximum concentration and area under the plasma concentration‐time curve with terminal elimination half‐life ranging from 29 to 40 days across the cohorts. The estimated absolute bioavailability after subcutaneous (SC) dosing was 51%. The average cerebrospinal fluid (CSF)‐to‐serum partition ratio was 0.12% (range 0.10% to 0.21%). Antidrug antibodies were detected transiently in <7% of participants at low titer, without effects on ABBV‐916 PK. This SAD study demonstrated a desirable safety, tolerability, and PK profile of ABBV‐916 after single‐dose administration in healthy participants.

These data supported further evaluation of multiple ABBV‐916 intravenous (IV) doses in patients with AD. It was hypothesized that ABBV‐916 would remove brain amyloid plaques in patients with early AD at significantly greater amounts than placebo, as measured by positron emission tomography (PET) at 24 weeks. Herein, we present results from a combined phase 1b/2 study that evaluated the safety and reduction in amyloid as measured by amyloid PET and PK, in patients with early AD treated with ABBV‐916.

## 2 METHODS

### 2.1 Study design and treatment

**FIGURE 1:**
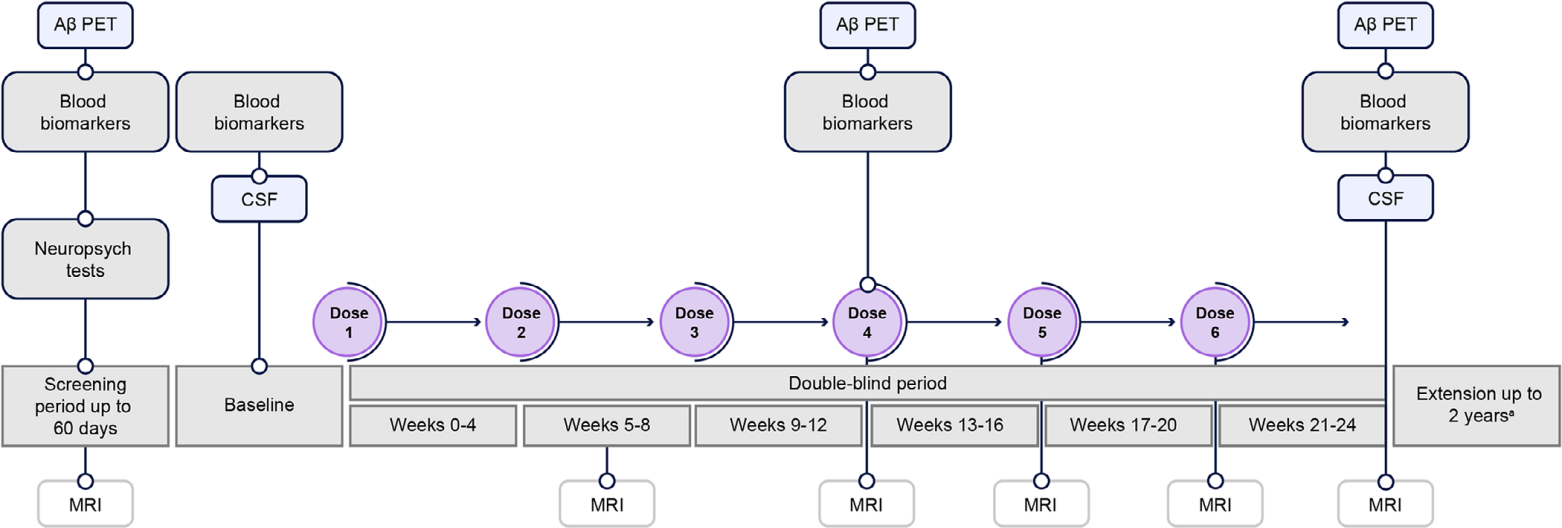
Study design during the double‐blind period. Aβ, amyloid beta; CSF, cerebral spinal fluid; MRI, magnetic resonance imaging; PET, positron emission tomography. ^a^Patients who chose not to participate in the 2‐year extension period completed a 16‐week follow‐up period.

The study was a combined phase 1b/2, global, multicenter, randomized, double‐blind, placebo‐controlled, multiple ascending dose study (NCT05291234). The study consisted of two stages: a multiple ascending dose stage (Stage A) and a dose expansion stage (Stage B). Both stages of the study were planned to consist of a 90‐day screening period, a 24‐week double‐blind period, and a 16‐week follow‐up safety period (Figure 1). Patients who completed the double‐blind period had the option of participating in a 2‐year extension period. All sponsor personnel with direct oversight or management of the trial, investigators, study site personnel, and patients remained blinded throughout the double‐blind period of the study.

In the multiple ascending dose stage, patients were randomly assigned (3:1) using interactive response technology to receive ABBV‐916 or placebo across six planned cohorts. The planned doses included cohort 1 (10 mg or placebo), cohort 2 (30 mg or placebo), cohort 3 (100 mg or placebo), cohort 4 (300 mg or placebo), cohort 5 (900 mg or placebo), and cohort 6 (3000 mg or placebo). For all cohorts, the study drug was administered intravenously every 4 weeks during the 24‐week double‐blind period for a total of six doses. For dose escalation decisions, the safety committee convened to review safety and PK data after a minimum of eight patients in a cohort had completed two doses and first safety magnetic resonance imaging (MRI) assessment (week 6) for each cohort. In addition, cumulative safety data were reviewed at each meeting.

**RESEARCH IN CONTEXT**

1. **Systematic review**: This study evaluated the efficacy and safety of ABBV‐916, a recombinant humanized immunoglobulin G1 monoclonal antibody, in adults with early‐stage AD. Current scientific literature was reviewed to contextualize the results of this study. The relevant publications are appropriately cited.

2. **Interpretation**: After 24 weeks, administration of ABBV‐916 doses of ≥300 mg every 4 weeks (Q4W) significantly reduced amyloid, as measured by PET. ABBV‐916 exhibited dose‐proportional PK, with few serious adverse events. Of the patients treated with ABBV‐916, 38.8% experienced an amyloid‐related imaging abnormality. Amyloid clearance rates and safety were comparable to those of currently approved anti‐amyloid therapies for AD.

3. **Future directions**: Although these data show meaningful amyloid clearance, ABBV‐916 did not demonstrate significant advantages over currently approved treatments; thus, further development was discontinued by the sponsor. The results of this study will add to the existing knowledge in the field.

Adjustments to the number of patients per cohort and planned doses were permitted per protocol. Before patients in cohort 6 received the study drug, the planned dose for that cohort was modified from 3000 to 2000 mg; following additional safety review, patients in cohort 6 who initially received 2000 mg were down‐titrated to 900 mg. Eligible participants in screening at the time of the down‐titration of cohort 6 were enrolled in an additional cohort, cohort 7 (900 mg or placebo).

Doses and regimens of concomitant medications for symptomatic AD therapy remained stable throughout the study unless changes were deemed medically necessary. The study was discontinued prior to initiation of the dose expansion stage due to strategic deprioritization of the program by the sponsor.

The study was conducted in accordance with the protocol, independent Ethics Committees/Institutional Review Boards (IRBs), International Council for Harmonisation guidelines, and all applicable federal and local regulations governing clinical trial conduct and ethical principles that originated in the Declaration of Helsinki. All patients or their legally authorized representative provided written informed consent before screening or the start of any study‐specific procedures. Approval of this study was obtained through a central IRB, Advarra (Columbia, MD, USA).

### 2.2 Patients

Patients were eligible for the study if they were adults aged 50 to 90 years, had no clinically significant findings for laboratory parameters (as assessed by the investigator), and met the 2018 National Institute on Aging‐Alzheimer's Association Research Framework Criteria for stage 3 or 4 AD. These criteria included a Mini‐Mental State Examination score of 20 to 28, a plasma amyloid probability score (C2N Diagnostics) value consistent with increased likelihood of positive amyloid PET, and an amyloid PET scan ≥ 37 Centiloids as assessed by florbetapir. Patients with MRI findings of vasogenic edema, at least four cerebral microhemorrhages, any area of superficial siderosis, any macrohemorrhage, or severe white matter disease were excluded from the study. Patients were ineligible if they had other clinically significant or unstable medical conditions or if they had a history that would make them an unsuitable candidate for the study, as determined by the investigator.

### 2.3 Assessments

The primary endpoints were safety, PK, and the change from baseline to week 24 in brain amyloid plaque deposition as measured by florbetapir PET. The study also assessed the change from baseline in blood‐based biomarkers, including Aβ40/Aβ42, p‐tau217, and glial fibrillary acidic protein (GFAP), as exploratory endpoints. Assessments of these biomarkers were performed at Fujirebio (Malvern, PA, USA) using the Lumipulse^®^ immunoassay platform. PK parameters included C_max_ (maximum serum concentration), T_max_ (time to C_max_), and area under the curve (AUC, total drug exposure over time) for the first and sixth doses, serum clearance (CL) after the sixth dose, and serum concentration at the end of each dosing interval (C_trough_). The CSF‐to‐serum ABBV‐916 concentration ratio was estimated to evaluate blood‐brain barrier penetration. Antidrug antibody (ADA) classification (positive or negative) and titers for any positive ADA samples were determined.

Safety was monitored throughout the study and included observation of AEs, clinical laboratory tests, vital signs, electrocardiograms, and the Columbia‐Suicide Severity Rating Scale. MRI was also performed to determine the incidence of ARIA‐E and ARIA‐H. ARIA severity was classified according to prespecified radiographic criteria (Table S1).

### 2.4 Statistical analysis

Each cohort was planned to consist of at least eight patients, with six receiving ABBV‐916 and two receiving placebo. For all analyses, patients were grouped according to actual treatment received. Patients receiving placebo from all cohorts were combined into a single placebo group; results from patients in cohort 5 (900 mg) and cohort 7 (900 mg) were combined for analysis. The sample size was determined to be adequate for assessing multiple ascending doses and for the probability that a given AE would not be observed in a 24‐week period. The primary analysis was conducted after all patients finished the double‐blind period. The full analysis set included all randomized patients who received ≥1 dose of the study drug during the double‐blind period and had a baseline amyloid PET assessment. The full analysis set was used for all efficacy and baseline analyses.

The change from baseline in amyloid Centiloid value at weeks 12 and 24 of the double‐blind period was analyzed using a mixed‐effects model for repeated measures (MMRM). The MMRM included fixed, categorical effects for treatment visit (week 12 or 24), treatment‐by‐visit interactions, with continuous fixed covariate for baseline amyloid Centiloid value. Amyloid negativity status (threshold < 24.1 Centiloids) was determined based on the last observed post‐baseline amyloid reduction compared with prespecified criteria, regardless of intercurrent events (e.g., discontinuation due to study drug, lost to follow‐up, or death). Summary statistics were calculated for each PK parameter for all patients who received ≥1 dose of study drug during the double‐blind period; analyses were performed on dose‐normalized C_max_, dose‐normalized AUC, dose‐normalized C_trough_, and β (elimination rate constant). The safety analysis included all patients who received ≥1 dose of study drug during the double‐blind period. Missing data due to intercurrent events were handled with hypothetical strategy.

### Data availability

AbbVie is committed to responsible data sharing regarding the clinical trials we sponsor. This includes access to anonymized, individual, and trial‐level data (analysis data sets), as well as other information (e.g., protocols, clinical study reports, synopses, or statistical analysis plans), as long as the trials are not part of an ongoing or planned regulatory submission.

These clinical trial data can be requested by any qualified researchers who engage in rigorous, independent, scientific research and will be provided following review and approval of a research proposal, Statistical Analysis Plan, and execution of a Data Use Agreement (DUA). Data requests can be submitted at any time after approval in the US and Europe and after acceptance of this manuscript for publication. For more information on the process or to submit a request, visit the following link: https://vivli.org/ourmember/abbvie/ then select “Home.”

## 3 RESULTS

### 3.1 Demographics and baseline characteristics

**FIGURE 2:**
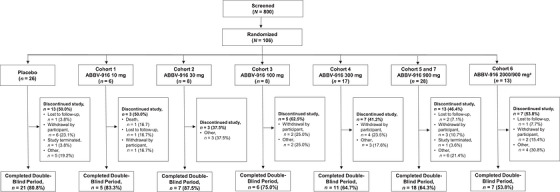
Patient disposition. Patients who discontinued study treatment are counted under each reason for discontinuation; thus, the sum of the counts given for the reasons for discontinuation may be greater than the overall number of discontinuations. ^a^The 2000‐mg cohort was down‐titrated to 900 mg from week 4 onward.

Participants were recruited from August 15, 2022, to May 9, 2024; the long‐term extension period is ongoing at the time of writing. Overall, 106 patients were randomized to receive ABBV‐916 (*n =* 80) or placebo (*n =* 26) (Figure 2). Of the 106 patients randomized, 75 completed the double‐blind period. About half of the patients in the study were female (placebo, 42.3%; ABBV‐916, 57.5%), and most were White (placebo, 96.2%; ABBV‐916, 88.8%); the mean (SD) age of patients receiving placebo was 76.4 (7.4) years and 76.2 (7.5) years in patients receiving ABBV‐916 (Table 1). In each cohort, ≥ 50% of patients were *APOE* ε4 carriers.

**TABLE 1:** Baseline demographics and characteristics.

Characteristic	Placebo (*n =* 26)	Cohort 1 10 mg (*n =* 6)	Cohort 2 30 mg (*n =* 8)	Cohort 3 100 mg (*n =* 8)	Cohort 4 300 mg (*n =* 17)	Cohort 5 and 7 900 mg (*n =* 28)	Cohort 6 2000 or 900 mg^a^ (*n =* 13)	Total (all ABBV‐916 doses) (*n =* 80)
Age, years, mean (SD)	76.4 (7.4)	75.2 (9.3)	77.0 (6.3)	79.1 (7.2)	76.4 (7.7)	76.8 (6.4)	72.8 (9.6)	76.2 (7.5)
Sex								
*Female*	11 (42.3)	4 (66.7)	4 (50.0)	4 (50.0)	11 (64.7)	14 (50.0)	9 (69.2)	46 (57.5)
*Male*	15 (57.7)	2 (33.3)	4 (50.0)	4 (50.0)	6 (35.3)	14 (50.0)	4 (30.8)	34 (42.5)
Race, *n* (%)								
*White*	25 (96.2)	5 (83.3)	7 (87.5)	8 (100)	16 (94.1)	23 (82.1)	12 (92.3)	71 (88.8)
*Black*	1 (3.8)	1 (16.7)	1 (12.5)	0	1 (5.9)	4 (14.3)	1 (7.7)	8 (10.0)
*Asian*	0	0	0	0	0	1 (3.6)	0	1 (1.3)
Ethnicity, *n* (%)								
*Hispanic or Latino*	6 (23.1)	3 (50.0)	2 (25.0)	1 (12.5)	3 (17.6)	6 (21.4)	4 (30.8)	19 (23.8)
BMI, mean (SD)	27.6 (3.9)	26.7 (6.5)	25.4 (3.0)	27.3 (4.8)	27.3 (4.0)	26.9 (5.1)	26.5 (4.0)	26.8 (4.5)
*APOE* 𝜀4 allele status, *n* (%)								
*ɛ2/ɛ2*	0	0	0	0	0	0	0	0
*ɛ2/ɛ3*	2 (7.7)	0	0	0	1 (5.9)	2 (7.1)	1 (7.7)	4 (5.0)
*ɛ2/ɛ4*	2 (7.7)	0	1 (12.5)	0	0	0	1 (7.7)	2 (2.5)
*ɛ3/ɛ3*	9 (34.6)	3 (50.0)	1 (12.5)	2 (25.0)	6 (35.3)	10 (35.7)	4 (30.8)	26 (32.5)
*ɛ3/ɛ4*	8 (30.8)	3 (50.0)	6 (75.0)	5 (62.5)	8 (47.1)	14 (50.0)	6 (46.2)	42 (52.5)
*ɛ4/ɛ4*	5 (19.2)	0	0	1 (12.5)	2 (11.8)	2 (7.1)	1 (7.7)	6 (7.5)
*APOE* 𝜀4 carrier status, n (%)								
*Positive*	15 (57.7)	3 (50.0)	7 (87.5)	6 (75.0)	10 (58.8)	16 (57.1)	8 (61.5)	50 (62.5)
*Negative*	11 (42.3)	3 (50.0)	1 (12.5)	2 (25.0)	7 (41.2)	12 (42.9)	5 (38.5)	30 (37.5)

*Note*: Patients were grouped by the first dose of ABBV‐916 received.

Abbreviation: BMI, body mass index.

^a^ The 2000‐mg cohort was down‐titrated to 900‐mg dose from week 4 onward.

### 3.2 Amyloid PET

**FIGURE 3:**
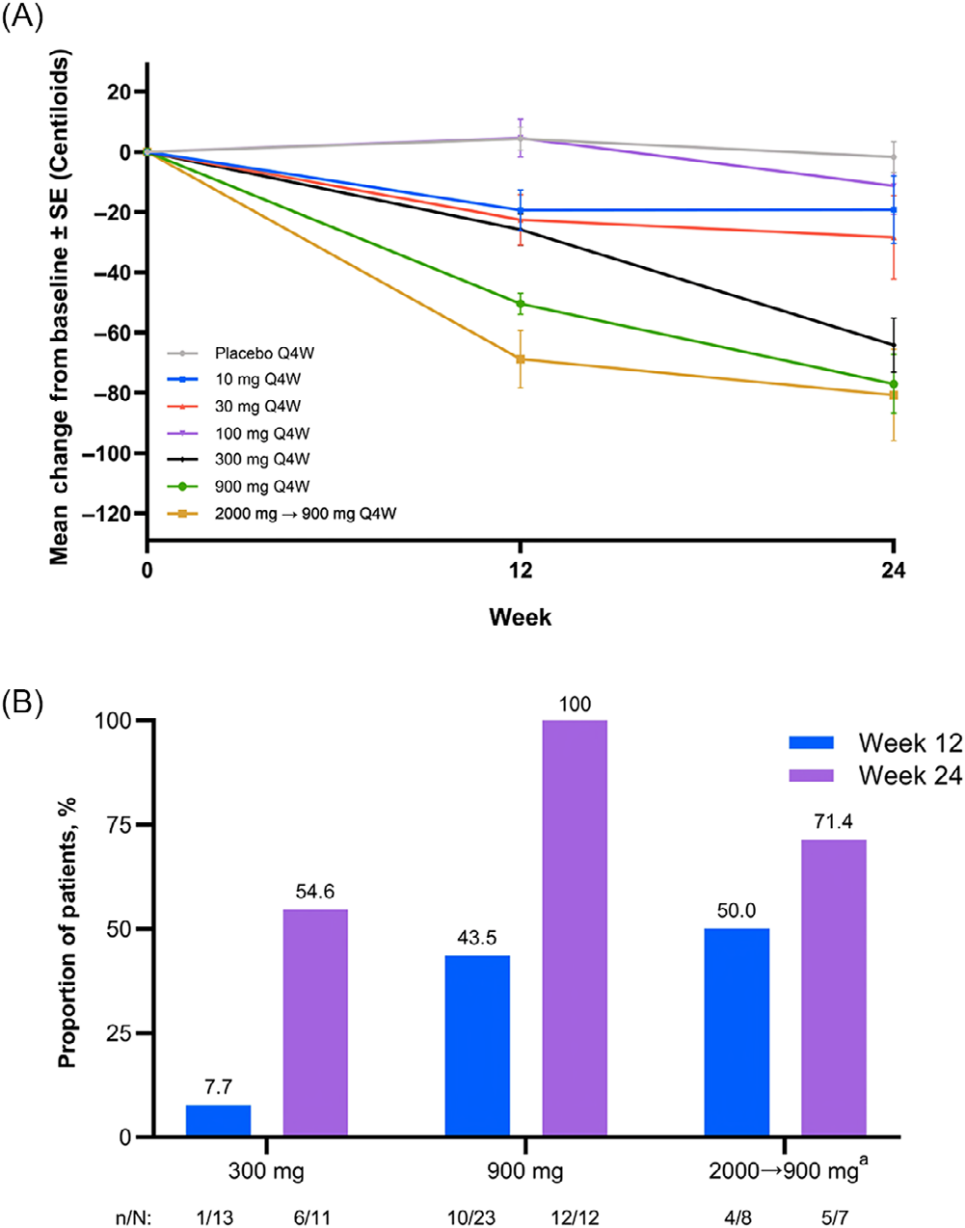
Dose‐dependent amyloid reduction after treatment with ABBV‐916. (A) Brain amyloid reduction (in Centiloids) was based on the actual treatment received; patients received treatment every 4 weeks, with the 2000‐mg cohort down‐titrated to 900 mg from week 4 onward. Mean baseline Centiloid values for each of the indicated treatment groups were as follows: placebo, *n =* 26, 97.9 Centiloids; 10 mg, *n =* 6, 83.3 Centiloids; 30 mg, *n =* 8, 102.9 Centiloids; 100 mg, *n =* 8, 94.4 Centiloids; 300 mg, *n =* 17, 102.8 Centiloids; 900 mg, *n =* 28, 85.2 Centiloids; 2000→900 mg, *n =* 13, 86.9 Centioids. N's for each group shown above at weeks 12 and 24 are as follows: placebo, week 12, *n =* 25; week 24, *n =* 40. 10 mg, week 12, *n =* 6; week 24, *n =* 7. 30 mg, week 12, *n =* 7; week 24, *n =* 7. 100 mg, week 12, *n =* 8; week 24, *n =* 6. 300 mg, week 12, *n =* 13; week 24, *n =* 11. 900 mg, week 12, *n =* 23; week 24, *n =* 12. 2000 mg → 900 mg, week 12, *n =* 8; week 24, *n =* 7. Patients receiving placebo from all cohorts were combined into a single placebo group. Wk, week. (B) Percentage of patients who achieved amyloid negativity by week 24. Amyloid negativity was defined as < 24.1 Centiloids; *n* is the number of patients with a non‐missing baseline value and at least 1 post‐baseline value. ^a^The 2000‐mg cohort was down‐titrated to 900 mg from week 4 onward (cohorts based on first dose received).

After 24 weeks of treatment, levels of brain amyloid plaque decreased following treatment with all doses of ABBV‐916 (Figure 3A). Most patients in cohort 6 who were randomized to active treatment received ≥2 doses of ABBV‐916 at 2000 mg before down‐titration to 900 mg, with one patient receiving one dose at 2000 mg before down‐titration to 900 mg. At 24 weeks, the MMRM analysis (excluding the 2000/900 mg cohort, cohort 6) showed that, compared with placebo, ABBV‐916 resulted in a statistically significant reduction in brain amyloid plaque levels from baseline at dose levels of 30 mg (*p* < 0.05), 300 mg (*p* < 0.0001), and 900 mg (*p* < 0.0001). The estimated difference from placebo at week 24 was −25.1 Centiloids, −61.9 Centiloids, and −83.7 Centiloids for the 30 mg, 300 mg, and 900 mg doses, respectively. By week 12, 7.7% of patients receiving ABBV‐916 300 mg, 43.5% of those receiving ABBV‐916 900 mg, and 50.0% of those receiving ABBV‐916 2000/900 mg (down‐titrated cohort) achieved amyloid negativity (Figure 3B). By week 24, 54.6% of patients receiving ABBV‐916 300 mg, 100% receiving ABBV‐916 900 mg, and 71.4% receiving ABBV‐916 2000/900 mg (down‐titrated cohort) achieved amyloid negativity. In contrast, those receiving 10, 30, or 90 mg ABBV‐916 achieved no more than 20% amyloid negativity at either week 12 or 24.

### 3.3 Biomarkers

To determine the changes in plasma biomarkers at the dose levels where ABBV‐916 reduces brain amyloid plaque, we compared plasma levels of Aβ 42/40, p‐tau217, and GFAP in patients who received ABBV‐916 at 300 mg and 900 mg versus that of patients in the placebo group. After 24 weeks of treatment, patients who received ABBV‐916 showed an increase from baseline in Aβ42/Aβ40 ratio (an increase of 15.3% and 15.7% for ABBV‐916 300 and 900 mg, respectively, versus a decrease of 2.3% for placebo), a decrease from baseline in p‐tau217 (a decrease of 23.8% and 29.8% for ABBV‐916 300 mg and 900 mg, respectively, vs an increase of 15% for placebo), and a decrease in GFAP (a decrease of 20.0% and 31.5% for ABBV‐916 300 mg and 900 mg, respectively, vs an increase of 11.9% for placebo) (Figure S1).

### 3.4 Pharmacokinetics

**FIGURE 4:**
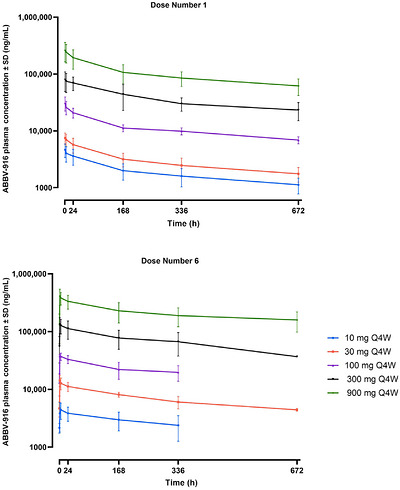
Serum concentration of ABBV‐916 over time. (Top) After the first dose. (Bottom) After the sixth dose. Q4W, every 4 weeks.

**FIGURE 5:**
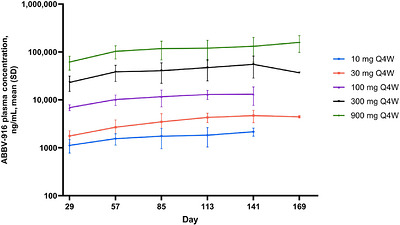
Mean trough concentration (C_trough_) of ABBV‐916 over the double‐blind period, starting with day 29. Q4W, every 4 weeks.

The data from cohort 6 (ABBV‐916 2000/900 mg) were not included in the PK analysis. The serum concentration of ABBV‐916 increased in a dose‐proportional manner after the first and sixth doses (Table 2; Figure 4). Likewise, during the double‐blind period, ABBV‐916 serum trough concentrations (C_trough_) demonstrated dose‐proportional increases over time (Figure 5), with C_trough_ reaching steady state by about 16 weeks (Figure 5). The ABBV‐916 CSF‐to‐serum concentration ratio was 0.2% to 0.4% after the sixth dose at doses ≥30 mg Q4W, with ABBV‐916 CSF concentrations at 10 mg Q4W below detectable levels. Positive ADAs were detected in 3.8% (3/80) of patients treated with ABBV‐916 during the 24‐week double‐blind period; of these, 67% (2/3) were treatment‐emergent. All patients who developed ADAs had low titers (10 is the lowest titer considered ADA positive); all ADA positivity was transient.

**TABLE 2:** Pharmacokinetic parameters of ABBV‐916 after IV administration.

Characteristic, Geometric Mean (% CV)	Cohort 1 10 mg (*n =* 5)	Cohort 2 30 mg (*n =* 7)	Cohort 3 100 mg (*n =* 8)	Cohort 4 300 mg (*n =* 16)	Cohort 5 and 7 900 mg (*n =* 27)
**After the 1st Dose**					
C_max_, µg/mL	4.5 (4.6, 27)	7.3 (7.5, 22)	30.9 (31.7, 26)	86.9 (88.8, 20)	257 (273, 36)
T_max_, h^a^	1 (1, 1)	1 (1, 4)	1 (1, 4)	1 (1, 24)	1 (1, 4)
AUC_tau_, µg*h/mL	1170 (1240, 31)	1880 (1950, 29)	7370 (7400, 10)	23,900 (24,700, 29)	65,700 (68,500, 28)
C_max_/dose, (µg/mL)/mg	0.4 (0.5, 27)	0.2 (0.3, 22)	0.3 (0.3, 26)	0.3 (0.3, 20)	0.3 (0.3, 36)
AUC_tau_/dose, (µg*h/mL)/mg	117 (124, 31)	62.6 (64.9, 29)	73.7 (74, 10)	79.6 (82.5, 29)	73 (76.1, 28)
**After the 6th Dose**					
C_max_, µg/mL	4.5 (4.7, 34)^b^	13.2 (13.5, 25)^c^	39.3 (39.5, 10)^d^	129 (135, 30)^e^	403 (420, 30)^f^
T_max_, h^a^	1 (1, 4)^b^	1 (1, 24)^c^	1 (1, 4)^d^	1 (1, 4)^e^	1 (0, 4)^f^
AUC_tau_, µg*h/mL	1560 (1680, 42)^b^	4400 (4460, 17)^c^	13,400 (13,800, 25)^c^	43,300 (46,100, 38)^e^	134,000 (142,000, 35)^g^
CL, mL/h	6.4 (6.9, 42)^b^	6.8 (6.9, 20)^c^	7.4 (7.6, 25)^c^	6.9 (7.3, 35)^e^	6.7 (7.1, 34)^g^
C_max_/dose, (µg/mL)/mg	0.4 (0.5, 34)^b^	0.4 (0.5, 25)^c^	0.4 (0.4, 10)^d^	0.4 (0.5, 30)^e^	0.4 (0.5, 30)^f^
AUC_tau_/dose, (µg*h/mL)/mg	156 (168, 42)^b^	147 (149, 17)^c^	134 (138, 25)^c^	144 (154, 38)^e^	149 (157, 35)^g^
CSF‐to‐serum ratio (%)^a^	0^j^	0.2 (0, 0.7)^k^	0.4 (0.2, 1.1)^h^	0.3 (0.1, 0.4)^l^	0.3 (0.2, 0.8)^e^

Abbreviations: AUC_tau_, area under the plasma‐concentration time curve due to a dosing interval; CL, clearance; C_max_, maximum serum concentration; CSF, cerebrospinal fluid; CV, coefficient of variation; h, hours; IV, intravenous; T_max_, time to C_max_.

^a^ Median (minimum, maximum).

^b^ *n =* 4.

^c^ *n =* 5.

^d^ *n =* 6.

^e^ *n =* 11.

^f^ *n =* 17.

^g^ *n =* 16.

^h^ *n = *7.

^i^ *n =* 15.

^j^ *n =* 3. All patients had CSF concentrations below the limit of quantification.

^k^ *n = *6. Three out of five patients had CSF concentrations below the limit of quantification.

^l^ *n = *13.

### 3.5 Safety

Across all ABBV‐916‐treated patients, 78.8% of patients reported ≥1 AE, 11.3% reported serious AEs (SAEs), and 20.0% discontinued due to AEs (Table 3). Most AEs were non‐serious and mild or moderate in severity. The most common AEs (≥10%) were ARIA‐E, ARIA‐H, headaches, falls, and urinary tract infections. Across all placebo‐treated patients, 69.2% of patients reported ≥1 AE, 0% reported SAEs, and none discontinued due to an AE. Throughout the double‐blind period, there were three infusion‐related reactions in patients receiving ABBV‐916 900 mg; all were non‐serious, and all patients recovered. There were no clinically meaningful changes observed in clinical laboratory tests, vital signs, electrocardiograms, or Columbia‐Suicide Severity Rating Scale scores. There were no deaths during the double‐blind period.

**TABLE 3:** Safety summary in the double‐blind period.^a^ ^.^

Characteristic, *n* (%)	Placebo (*n =* 26)	Cohort 1 10 mg (*n =* 6)	Cohort 2 30 mg (*n =* 8)	Cohort 3 100 mg (*n =* 8)	Cohort 4 300 mg (*n =* 17)	Cohort 5 and 7 900 mg (*n =* 28)	Cohort 6 2000 or 900 mg^b^ (*n =* 13)	Total (all ABBV‐916 doses) (*n =* 80)
TEAEs								
*Any*	18 (69.2)	5 (83.3)	2 (25.0)	3 (37.5)	16 (94.1)	25 (89.3)	12 (92.3)	63 (78.8)
*Severe*	0	1 (16.7)	0	0	4 (23.5)	3 (10.7)	1 (7.7)	9 (11.3)
*Serious*	0	1 (16.7)	0	0	4 (23.5)	3 (10.7)	1 (7.7)	9 (11.3)
Infusion‐related reactions	0	0	0	0	0	3 (10.7)	0	3 (3.8)
TEAEs resulting in treatment discontinuation	0	0	1 (12.5)	2 (25.0)	4 (23.5)	5 (17.9)	4 (30.8)	16 (20.0)
TEAEs in ≥5% of patients								
*ARIA‐E*	0	0	1 (12.5)	2 (25.0)	5 (29.4)	11 (39.3)	8 (61.5)	27 (33.8)
*ARIA‐H*	0	0	1 (12.5)	2 (25.0)	6 (35.3)	4 (14.3)	7 (53.8)	20 (25.0)
*Headache*	1 (3.8)	0	0	1 (12.5)	2 (11.8)	6 (21.4)	3 (23.1)	12 (15.0)
*Fall*	0	2 (33.3)	0	0	3 (17.6)	2 (7.1)	1 (7.7)	8 (10.0)
*UTI*	3 (11.5)	1 (16.7)	0	0	1 (5.9)	5 (17.9)	1 (7.7)	8 (10.0)
*Dizziness*	0	0	1 (12.5)	0	1 (5.9)	2 (7.1)	2 (15.4)	6 (7.5)
*Nausea*	0	0	1 (12.5)	0	0	2 (7.1)	3 (23.1)	6 (7.5)
*COVID‐19*	1 (3.8)	0	0	0	1 (5.9)	4 (14.3)	0	5 (6.3)
*Hypertension*	2 (7.7)	1 (16.7)	0	0	1 (5.9)	3 (10.7)	0	5 (6.3)
*Upper respiratory tract infection*	0	0	0	0	1 (5.9)	3 (10.7)	0	4 (5.0)
ARIA‐E by *APO* ɛ4 status, n/N (%)								
*Non‐carrier*	0/11	0/3	0/1	0/2	2/7 (28.6)	2/12 (16.7)	1/5 (20.0)	5/30 (16.7)
*Heterozygous carrier*	0/10	0/3	1/7 (14.3)	1/5 (20.0)	2/8 (25.0)	7/14 (50.0)	6/7 (85.7)	17/44 (38.6)
*Homozygous carrier*	0/5	0/0	0/0	1/1 (100)	1/2 (50.0)	2/2 (100)	1/1 (100)	5/6 (83.3)
ARIA‐H by *APO* ɛ4 status, *n*/*N* (%)								
*Non‐carrier*	0/11	0/3	0/1	0/2	2/7 (28.6)	1/12 (8.3)	1/5 (20.0)	4/30 (13.3)
*Heterozygous carrier*	0/10	0/3	1/7 (14.3)	1/5 (20.0)	4/8 (50.0)	2/14 (14.3)	5/7 (71.4)	13/44 (29.5)
*Homozygous carrier*	0/5	0/0	0/0	1/1 (100)	0/2	1/2 (50.0)	1/1 (100)	3/6 (50.0)
ARIA‐E								
*Symptomatic*	0	0	0	0	1 (5.9)	4 (14.3)	3 (23.1)	8 (10.0)
*Asymptomatic*	0	0	1 (12.5)	2 (25.0)	4 (23.5)	7 (25.0)	5 (38.5)	19 (23.8)
ARIA‐H								
*Microhemorrhage*	0	0	1 (12.5)	2 (25.0)	6 (35.3)	4 (14.3)	7 (53.8)	20 (25.0)
*Symptomatic microhemorrhage*	0	0	0	0	1 (5.9)	1 (3.6)	2 (15.4)	4 (5.0)
*Superficial siderosis*	0	0	1 (12.5)	2 (25.0)	3 (17.6)	1 (3.6)	4 (30.8)	11 (13.8)
*Symptomatic superficial siderosis*	0	0	0	0	1 (5.9)	1 (3.6)	1 (7.7)	3 (3.8)
*Macrohemorrhage*	0	0	0	0	0	0	0	0

Abbreviations: ARIA, amyloid‐related imaging abnormalities; ARIA‐E, amyloid‐related imaging abnormalities‐edema; ARIA‐H, amyloid‐related imaging abnormalities‐hemosiderin; TEAE, treatment‐emergent adverse events; UTI, urinary tract infection.

Patients were counted once in each row within a column, regardless of the number of events they may have had.

^a^ Summary includes all patients who received ≥1 dose of study drug or placebo; patients in ABBV‐916 cohorts were grouped by first dose received.

^b^ The 2000‐mg cohort was down‐titrated to 900‐mg dose from week 4 onward.

The incidences of ARIA‐E and ARIA‐H in patients treated with ABBV‐916 across all dose levels were 33.8% and 25.0%, respectively. Most of the ARIA‐E and ARIA‐H cases occurred at or within 12 weeks of treatment initiation. No patient in the placebo group experienced any ARIA‐E or ARIA‐H. Of the 27 ABBV‐916‐treated patients with ARIA‐E, 8/27 (29.6%) experienced symptomatic ARIA‐E; 2 patients had symptoms that were considered serious (cohort 4), and five patients with symptomatic ARIA‐E discontinued treatment. Of the 20 ABBV‐916‐treated patients with ARIA‐H, all 20 experienced cerebral microhemorrhage, and 4/20 (20.0%) were symptomatic; 11/20 (55.0%) experienced superficial siderosis, and 3/20 (15.0%) showed symptoms; no patients experienced macrohemorrhage. None of the ARIA‐H events were considered serious, and four patients with symptomatic ARIA‐H discontinued treatment. There appeared to be a dose‐dependent increase in patients experiencing ARIA events.

Of the 50 *APOE* ε4‐positive ABBV‐916‐treated patients, 22/50 (44.0%) and 16/50 (32.0%) experienced an ARIA‐E or ARIA‐H event, respectively. Of the 30 *APOE* ε4‐negative ABBV‐916‐treated patients, 5/30 (16.7%) and 4/30 (13.3%) experienced an ARIA‐E or ARIA‐H event, respectively. Of the six patients who were treated with ABBV‐916 and were *APOE* ε4 homozygous, 5/6 (83.3%) experienced an ARIA event; of the 44 *APOE* ε4 heterozygous ABBV‐916‐treated patients, 19/44 (43.2%) experienced an ARIA event.

## 4 DISCUSSION

Findings from this phase 1b/2 study show that ABBV‐916, particularly at doses ≥300 mg, reduced amyloid in a time‐ and dose‐dependent manner over 24 weeks of treatment, consistent with its proposed mechanism of action. PK exposures were dose‐proportional across all tested doses, with negligible ADA rates in patients. ABBV‐916 was generally well tolerated; most AEs experienced by patients were non‐severe and non‐serious and did not require treatment discontinuation.

While preclinical studies of ABBV‐916 did not identify significant microhemorrhage‐related concerns^13^ and the phase 1 studies of ABBV‐916 demonstrated a favorable safety profile in healthy volunteers,^14^ the lack of amyloid burden in these younger, healthy adults may not be generalizable to the current trial population who were between the ages of 50 and 90 years with stage 3 or 4 AD. For these reasons, while the lowest dose used in the phase I SAD study with healthy adults was 100 mg, subtherapeutic doses of 10 mg and 30 mg were selected for initial evaluation as a conservative approach in this multiple ascending dose study.^15^ The safety committee reviewed safety, efficacy, and PK data for each cohort prior to each dose escalation and ad hoc, and assessed cumulative safety data across the entire program, thereby conducting a benefit‐risk assessment at each meeting. The decision to reduce the cohort 6 dose escalation from approximately three‐fold to approximately two‐fold was informed by this process. The reduction of cohort 6 dosing from 2000 mg to 900 mg after week 4 was arrived at in a similar fashion, due to the observed earlier onset of ARIA‐E and ARIA‐H.

Amyloid reduction observed in this study with ABBV‐916 900 mg at 12 weeks (mean change: −50.4 Centiloids) and 24 weeks (−77.0 Centiloids) was numerically comparable to current disease‐modifying AD treatments.^16^, ^17^ The proportion of amyloid negativity at 24 weeks was 100% in the 900 mg combined cohorts 5 and 7, and it was 71.9% in cohort 6. ABBV‐916's amyloid negativity rate is comparable to donanemab's 40% amyloid negativity rate at 24 weeks and up to 67.8% at 76 weeks.^17^ Although the down‐titrated 2000/900 mg cohort achieved a lower rate of amyloid negativity at week 24 (71.4%; 5/7) compared to the 900 mg cohort (100%; 12/12), despite greater overall drug exposure, this finding should be interpreted with caution, given the small sample size. With small sample sizes, estimates of amyloid negativity are highly susceptible to individual‐level variability such as baseline amyloid burden and the timing of down‐titration relative to the trajectory of amyloid clearance.

The overall safety profile observed in this study (including ARIA incidence) was comparable to that of currently approved anti‐amyloid therapies.^16^, ^17^, ^18^, ^19^, ^20^ While targeting Aβ peptides bearing a pyroglutamate residue at amino acid position 3 (AβpE3) might be expected to reduce ARIA events, given that AβpE3 plaques are less abundant in the cerebral vasculature, the promising safety of ABBV‐916 in preclinical studies were not borne out; 33.8% of the ABBV‐916‐treated patients experienced an ARIA‐E event, and 25.0% experienced an ARIA‐H event in this study. Rates of ARIA in this study among *APOE* ε4 carriers, particularly for those homozygous for the gene, were elevated. Although the sample size of *APOE* ε4 carriers is small, the finding is consistent with the role of *APOE* ε4 genotype as a risk factor for ARIA, underscoring the need for genetic screening prior to treatment initiation.^21^ In addition to *APOE* ε4 carrier status, studies have shown that the incidence of ARIA‐E seemingly increases with the dose of anti‐amyloid treatment, an effect not as apparent when considering the rates of ARIA‐H events.^12^ In the current study, the incidence of ARIA overall increased with both higher doses of ABBV‐916 and the number of *APOE* ε4 alleles. These findings suggest that the selective targeting of modified amyloid species may not be enough to circumvent ARIA risk.

Furthermore, discontinuation rates were higher in cohorts receiving doses that were associated with greater amyloid clearance, suggesting decreased tolerability at exposure levels that may be required for clinically meaningful efficacy. The balance between therapeutic benefit and tolerability is an important consideration for treatment and highlights the need for further investigation to identify dosing levels and regimens that optimize amyloid reduction while minimizing safety risks such as ARIA‐related events.

### 4.1 Limitations

As this study was limited by a small sample size and short duration, results should be interpreted with caution. The study was discontinued prior to dose expansion, a critical factor that limits any potential conclusions regarding optimal dosing and long‐term safety. The study population was predominantly White, which limits the generalizability of the efficacy and safety findings to more diverse populations. Given that *APOE* ε4 allele frequency, baseline amyloid burden, and cerebrovascular risk profiles differ across racial and ethnic groups,^22^, ^23^ future studies should prioritize inclusive recruitment strategies to ensure findings are broadly applicable to all at‐risk populations. Additionally, interruptions in dosing and early discontinuation due to reasons such as adverse events may have introduced confounding variables that interfere with a complete assessment of the full effectiveness of ABBV‐916 in amyloid clearance. The study was also limited by the sponsor's decision not to advance the program due to business considerations, as the efficacy and safety profiles were not substantially different from those of currently approved therapies. Therefore, certain opportunities could not be further explored, such as evaluating whether ARIA rates would remain similar in a larger study population or whether dose titration or intermediate doses would mitigate some of the ARIA risks. That said, the study design would have easily enabled an operationally seamless transition to a phase 2 study. Another strength of this study design was the use of blood‐based biomarkers during the screening phase. Blood‐based biomarkers were used to screen out patients who had a low likelihood of amyloid accumulation, preventing the need for further screening procedures and minimizing patient burden.

### 4.2 Conclusion

Data from this phase 1b/2 study suggest that, while positive, the efficacy and safety profiles of ABBV‐916 in early AD are generally comparable to those of currently approved anti‐amyloid immunotherapies, with no clear evidence of greater benefits or other meaningful differentiation.

## AUTHOR CONTRIBUTIONS

Shau Yu Lynch contributed to the research project conception and design, data acquisition, data interpretation, drafting, review and critique of the manuscript, and approval of the final manuscript draft submitted for publication. Yamin Wang contributed to the research project conception and design, data acquisition, data analysis, data interpretation, drafting, review and critique of the manuscript, and approval of the final manuscript draft submitted for publication. Deli Wang contributed to the research project conception and design, data analysis, data interpretation, drafting, review and critique of the manuscript, and approval of the final manuscript draft submitted for publication. Sagar S. Bachhav contributed to the research project conception and design, data acquisition, data analysis, data interpretation, drafting, review and critique of the manuscript, and approval of the final manuscript draft submitted for publication. Hao Xiong contributed to the research project conception and design, data acquisition, data interpretation, drafting, review and critique of the manuscript, and approval of the final manuscript draft submitted for publication. Joey Boiser, Edwin Stage, Anthony W. Bannon, Ole Graff and Hana Florian each contributed to the research project conception and design, data interpretation, drafting, review and critique of the manuscript, and approval of the final manuscript draft submitted for publication.

## CONFLICT OF INTEREST STATEMENT

Shau Yu Lynch, Yamin Wang, Deli Wang, Hao Xiong, Joey Boiser, Edwin Stage, Anthony W. Bannon, Ole Graff, Hana Florian, Angela Hadsell, and Anita Preininger are employees of AbbVie Inc. and may hold AbbVie stock and/or stock options. Sagar S. Bachhav is a former AbbVie employee, currently employed by Merck, and may hold AbbVie stock. Author disclosures are available in the Supporting Information.

## CONSENT STATEMENT

Before engaging in any study activities, all participants and study partners provided written informed consent.

## Supporting information



Supporting Information
