# Supplementary material for: A randomized phase 1b/2 trial of ABBV‐916 in adults with early Alzheimer's disease
Source: Alzheimers Dement. 2026 Aug 7;22(8):e71715. doi: 10.1002/alz.71715 (PMC13448778; doi:10.1002/alz.71715)
Supplement: Supplementary file 1 [file ALZ-22-e71715-s001.docx]

| **Supplementary Table 1. ARIA Severity Classification Criteria** | | | |
| --- | --- | --- | --- |
| **ARIA Type** | **Radiographic Severity** | | |
|  | **Mild** | **Moderate** | **Severe** |
| ARIA-E | Mild FLAIR hyperintensity  < 5 cm involving 1 region,  confined to sulcus and/or  cortex/subcortex white  matter | FLAIR hyperintensity  5 to 10 cm or > 1 region  of involvement, each  < 10 cm | FLAIR hyperintensity  > 10 cm,  ≥ 1 separate or independent  regions of involvement  may be noted |
| ARIA-H microhemorrhage | ≤ 4 treatment-emergent  microhemorrhages | 5 to 9 treatment-emergent  microhemorrhages | ≥ 10 treatment-emergent  microhemorrhages |
| ARIA-H superficial siderosis | 1 focal area | 2 focal areas | > 2 focal areas |
| ARIA, amyloid-related imaging abnormalities; ARIA-E, ARIA-edema; ARIA-H, ARIA-hemorrhage; FLAIR, fluid-attenuated inversion recovery. | | | |

**Supplementary Figure 1. Mean change from baseline in biomarkers.**
Aβ, amyloid beta; GFAP, glial fibrillary acidic protein; p-tau, phosphorylated tau protein. Units for p-tau217 and GFAP baseline values are given in pg/mL.
